# Evaluation of the diagnostic performance of infrared imaging of the breast: a preliminary study

**DOI:** 10.1186/1475-925X-9-3

**Published:** 2010-01-07

**Authors:** Jane Wang, King-Jen Chang, Chin-Yu Chen, Kuo-Liong Chien, Yuh-Show Tsai, Yuh-Ming Wu, Yu-Chuan Teng, Tiffany Ting-Fang Shih

**Affiliations:** 1 Department of Medical Imaging, National Taiwan University Hospital, 7 Chung-Shan South Road, Taipei 100, Taiwan; 2 Department of Radiology, College of Medicine, National Taiwan University, No 1, Section 1, Jen-Ai Road, Taipei 100, Taiwan; 3 Department of Surgery, National Taiwan University Hospital, 7 Chung-Shan South Road, Taipei 100, Taiwan; 4 Department of Radiology, Chi-Mei Medical Center, 901 Chung Hwa Road, Yung-Kang City, Tainan 710, Taiwan; 5 Institute of Preventive Medicine, College of Public Health, National Taiwan University, 17 Hsu-Chow Road, Taipei 100, Taiwan; 6 Department of Biomedical Engineering, Chung Yuan Christian University, 200 Chung Pei Road, Chung-Li 32023, Taiwan; 7 Department of Biomedical Engineering, National Taiwan University Hospital, 7 Chung-Shan South Road, Taipei 100, Taiwan

## Abstract

**Background:**

The study was conducted to investigate the diagnostic performance of infrared (IR) imaging of the breast using an interpretive model derived from a scoring system.

**Methods:**

The study was approved by the Institutional Review Board of our hospital. A total of 276 women (mean age = 50.8 years, SD 11.8) with suspicious findings on mammograms or ultrasound received IR imaging of the breast before excisional biopsy. The interpreting radiologists scored the lesions using a scoring system that combines five IR signs. The ROC (receiver operating characteristic) curve and AUC (area under the ROC curve) were analyzed by the univariate logistic regression model for each IR sign and an age-adjusted multivariate logistic regression model including 5 IR signs. The cut-off values and corresponding sensitivity, specificity, Youden's Index (Index = sensitivity+specificity-1), positive predictive value (PPV), negative predictive value (NPV) were estimated from the age-adjusted multivariate model. The most optimal cut-off value was determined by the one with highest Youden's Index.

**Results:**

For the univariate model, the AUC of the ROC curve from five IR signs ranged from 0.557 to 0.701, and the AUC of the ROC from the age-adjusted multivariate model was 0.828. From the ROC derived from the multivariate model, the sensitivity of the most optimal cut-off value would be 72.4% with the corresponding specificity 76.6% (Youden's Index = 0.49), PPV 81.3% and NPV 66.4%.

**Conclusions:**

We established an interpretive age-adjusted multivariate model for IR imaging of the breast. The cut-off values and the corresponding sensitivity and specificity can be inferred from the model in a subpopulation for diagnostic purpose.

**Trial Registration:**

NCT00166998.

## Background

Infrared (IR) imaging of the breast, also known as breast thermography, is a non-invasive, painless examination which does not expose the subject to ionizing radiation, and is mainly a test of physiologic response of the breast findings [1-6]. It is based on the mechanism that the skin temperature overlying a malignancy is higher than skin overlying normal breast tissue. This is due to increasing infrared radiation and is most likely caused by elevated blood flow, metabolic activity, and angiogenesis at and around the lesion site [2,5]. IR imaging has been used for breast cancer detection since the 1970s [1,2]. A nationwide study, Breast Cancer Detection Demonstration Projects (BCDDP) launched in 1973, investigated breast cancer screening by clinical breast examination, mammography and IR imaging. However, IR imaging was dropped at an early stage of the project due to unsatisfactory results [1-4]. This may have been due to technical difficulties, widely variable and subjective interpretation among image readers, unacceptably high false-positive and false-negative rates, and no direct aid for spatial localization of surgery [1-4]. However, abnormal findings on IR imaging of the breast were reported to be a risk factor and useful prognostic predictor for breast cancer, and IR imaging can also be an aid in the differential diagnosis of benign from malignant tumors [1,3,6-10]. The aforementioned values can be facilitated by modern computerized IR technology [1,3,6,7,10-12]. On the other hand, the diagnostic criteria varied among studies and the diagnostic performance, including sensitivity and specificity, also varied. Herein, we investigated the diagnostic performance of computerized breast IR imaging using an integrated interpretative model for breast IR imaging.

## Methods

### Patients

The study was approved by the Institutional Review Board of our hospital, and all study participants signed informed consents before the study. We enrolled 276 women (ages 17-81 years, mean = 50.8 years, SD = 11.8 years, median 50 years) who were scheduled to undergo excisional biopsy for suspicious findings on mammograms or ultrasound, or both. Patients with probably benign findings on mammography or ultrasound but received excisional biopsy due to surgeons' concerns were also included. IR imaging of the breast was done 1 day before surgery. Before enrolling subjects into this study, we excluded women who had a past history of breast surgery or chest irradiation, or systemic chemotherapy. The participants had to refrain from smoking, alcohol drinking, vigorous exercise, and application of lotion to the breasts within 4 hours before the procedure. Patients who received fine needle aspiration within 2 days or core needle breast biopsy within 2 weeks or any of who received vacuum-assisted breast biopsy before the study were also excluded.

### Procedures

Computerized IR examination was done by two trained female radiological technicians using a medical thermographic system (ATIR-M301 Thermal Imaging System, Associated Technology Corporation, Chongqing, Sichuan, PROC), which was an uncooled micro-bolometer with focal plane array detector, and the image matrix size was 320 × 240 with 14-bit depth, the pixel size was 45 × 45 μm with the response wave length 8-12 μm, and the temperature resolution was less than 0.1°C. The procedure was done in a temperature controlled room maintained between 23 and 25°C. Each participant was asked to disrobe and sit on a chair in an erect position, with hands over the head, sitting a distance about 2.5 meters away from the IR camera. After a total of 15 minutes rest, frontal, two true lateral (left and right lateral) and two oblique (left and right oblique) views of IR images were taken.

### Imaging processing and interpretation

The images were viewed with a dedicated software program (M301-APP-V2.0, Associated Technology Corp., Chongqing, Sichuan, PROC) with manual brightness and contrast adjustment, and were displayed with either a gray-scale or a preset colored-scale.

A radiological technician and a radiologist (first radiologist) marked the lesion location and size of the lesions of concern, based on conventional imaging modalities including mammography, ultrasound, or both. They recorded the above information for each lesion on a sheet for the reference of the interpreting radiologists. Other two radiologists (second and third radiologists) were assigned to interpret the IR images; each read half of the cases. They interpreted the assigned IR images based only on the information of the above-mentioned sheet. The detailed mammographic and ultrasonographic, final pathologic findings of the study cases were only known to the first radiologist and were not available to the two IR imaging readers. The two radiologists who interpreted the IR images were both specialized in the field of breast imaging for more than 10 years.

**Figure 1:**
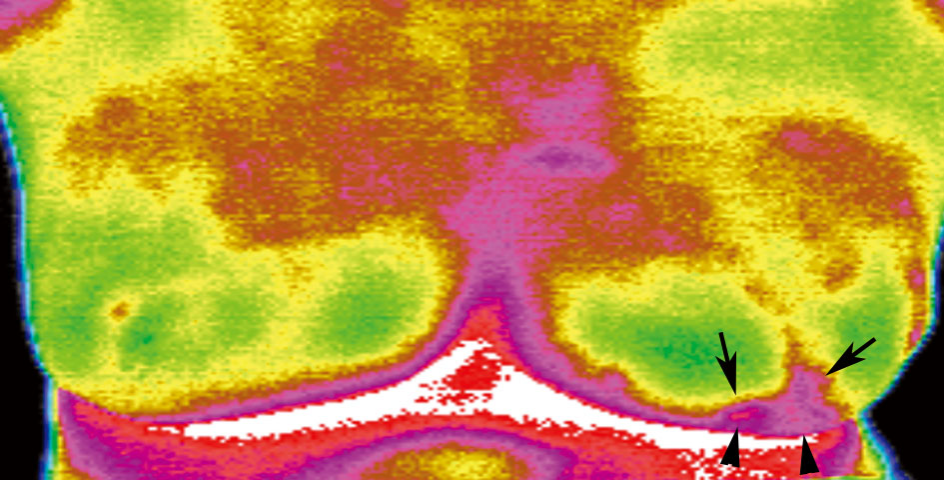
**A 76-year-old woman with left breast cancer**. IR imaging reveals focal increased surface temperature (positive IR1 sign with dT = 1.5°C compared with the contralateral mirror image site; positive IR2 sign with dT = 2°C compared with the remaining breast tissue at the ipsilateral side), abnormal vascular pattern (IR3 signs including closed vascular pattern, and vascular completeness) (arrows) and asymmetric vascular pattern (IR5 sign), and subtle focal bulging with back heat (IR4 sign) in left lower breast (arrowheads). Surgical pathological finding revealed a 4 cm infiltrating ductal carcinoma.

**Figure 2:**
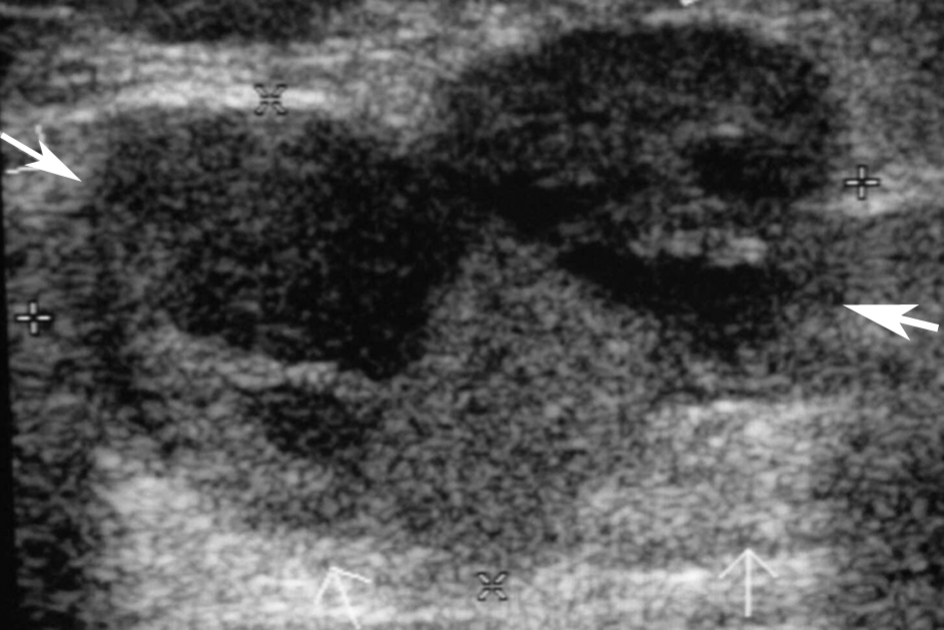
**The corresponding breast ultrasound of the patient in Figure 1 shows a lobular mass at left lower breast, measuring about 3.1 × 2.1 cm in diameter with heterogeneous echogenicity (arrows)**.

**Figure 3:**
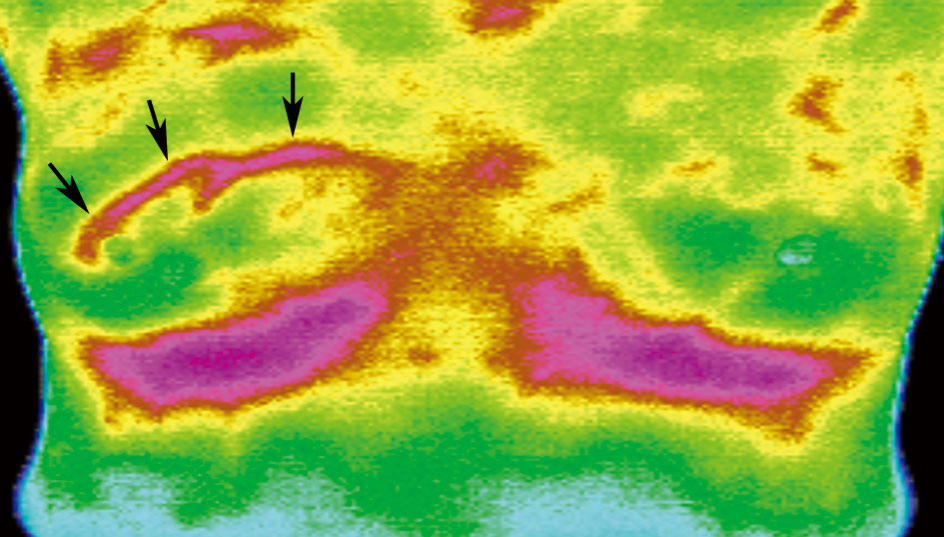
**A 48-year-old woman with right breast cancer**. IR imaging shows abnormally increased focal surface temperature (positive IR1 sign with dT = 1.2°C compared with the contralateral mirror image site; positive IR2 sign with dT = 2°C compared with the remaining breast tissue in the ipsilateral breast), abnormal vascular pattern (IR3 signs including bifurcated vascular pattern, transverse vascular pattern, vascular completeness) and an asymmetric vascular pattern (IR5) in the right upper breast (arrows). The ln(OD) value for this finding scored by the interpreting radiologist was: -5.463+0.0872(48)+0.3783(1)+1.9157(0)+0.1728(1)+0.1578(3)+1.0278(0)+1.0363(1) = 0.7834, which is higher than the most optimal cut-off point (0.30) we selected in Table 4, and this is test-positive based on this cut-off point. (The radiologist scored the IR1 scale for this lesion as 1, therefore, the IR1_A _= 1 and IR1_B _= 0).

The interpreting radiologists read the IR images based only on the findings at the lesion sites of concern and scored the findings according to the five independently diagnostic IR signs modified from the Ville Marie Infrared (IR) grading scale [7] and other reported literature [13-15]. The readers then recorded individual scores for each diagnostic IR sign for each lesion. We defined the IR signs as follows (Figures 1, 2, 3, &4):

**Figure 4:**
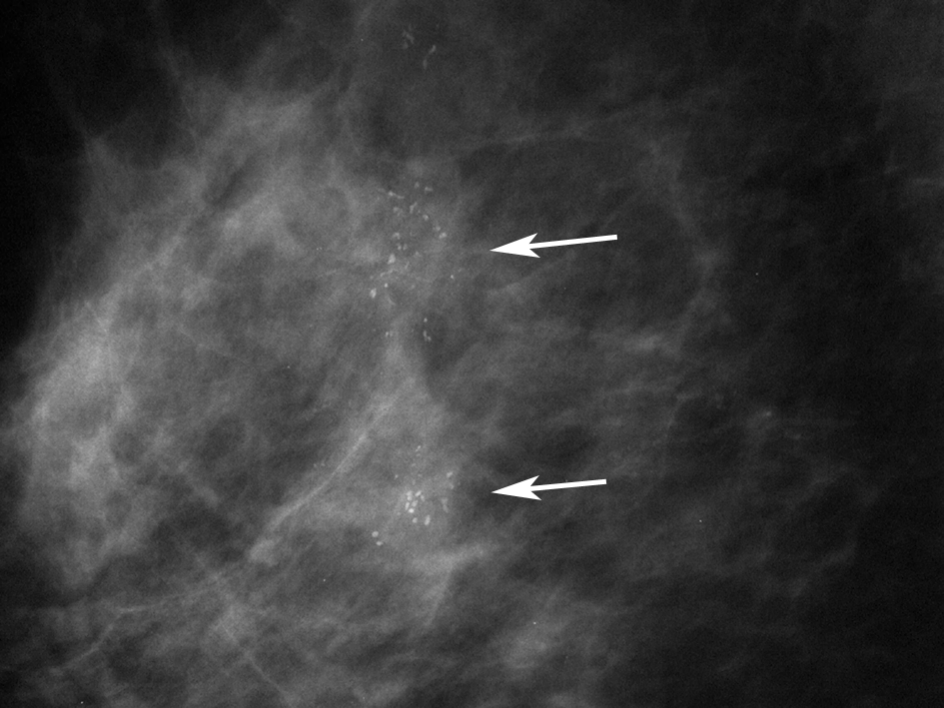
**The right magnified mammogram of the patient in Figure 3 shows segmental pleomorphic microcalcifications in right upper breast (arrows)**.

IR1: a difference in surface temperature (dT) at the lesion site from that at the mirror image site on the contralateral breast; the IR1 scale was scored as 0 for dT <=1°C, as 1 for 1°C < dT <=2°C, and as 2 for dT > 2°C.

IR2: the dT between the lesion site and the rest of the normal breast tissue of the ipsilateral breast; the IR2 scale was scored as 0 for dT <= 1°C, and as 1 for dT > 1°C.

IR3: a combination of 8 various abnormal vascular patterns, including star vessel, inverted V vessel, fragmented vessel, closed vessel, vascular completeness, pointed or bifurcated vessels, moa-moa vascular pattern, or transverse vascular pattern [14]. Of them, the star vessels indicated vessels with radiating pattern and star shape; the fragmented vessels indicated that fragmented vascular anarchy in a localized area of the breast; closed vessel indicated vascular anarchy arranged in a closed pattern without evident branching; pointed or bifurcated vessels indicated vessels with pointed or bifurcated ends; moa-moa sign indicated a focal area of abnormal vascular pattern with irregular and engorged vascular branching with its shape mimicking a moa; transverse vascular pattern indicated a vessel which traverses part of the breast with a relatively somewhat horizontal or transverse orientation [14]. The presence of any of the above signs was scored as 1 and the absence of such signs as 0. The sum of the eight signs was the score for the IR3 sign, which ranged from 0 to 8 and was treated as a continuous variable.

IR4: an edge sign or bulge sign backed by heat, indicating loss of smooth contour of part of the breast due to skin retraction or bulging caused by a breast tumor [16], the IR4 was scored as 0 when the sign was absent and as 1 when the sign was present.

IR5: the presence of an asymmetric or heterogeneous vascular pattern at and around the lesion site, when the contralateral breast did not reveal such a pattern. The IR5 scale was scored as 0 if the sign was absent and 1 if the sign was present.

### Statistical Analysis

We analyzed the correlation of the diagnostic IR signs with the final disease status of the lesions at surgery using univariate and multivariate logistic regression analyses by SAS software (SAS version 9.00, SAS Institute, Cary, NC, USA) and SPSS software (SPSS version 16.0, SPSS Inc., Chicago, IL, USA). We categorized the final disease status as a dichotomous variable, that is, benign (including high risk lesions) or malignant. The high-risk lesions in our study included ADH (atypical ductal hyperplasia), ALH (atypical lobular hyperplasia), and papillary lesions.

For the univariate logistic regression analysis, we investigated the association of each individual IR sign with the final disease status. For the multivariate regression model, we put the five IR signs of each lesion into the model with age adjustment. The AUC (area under the ROC curve) values of the ROC (receiver operating characteristic) curves for each IR sign from the univariate model and for the age-adjusted multivariate model were estimated in order to investigate the diagnostic performance of the IR imaging. The Hosmer and Lemeshow test was applied to inspect the goodness-of-fit of the age-adjusted multivariate model, and a non-significant *p *value (>0.05) implies that the model is a fitted model. The age-adjusted multivariate model was presented as shown below:

Where ln indicated the natural logarithm. The P value indicated the predicted probability of a lesion being malignant estimated from the regression model. Therefore, the P/(1-P) ratio indicated the odds (OD), that is, the ratio of the probability of being malignant to being benign for a given lesion. α is the intercept of the model. However, since IR1 is a trichotomous categorical variable, we re-coded the IR1 sign as a dummy variable for computations in logistic regression. That is:

if the IR1 scale of a given lesion was 0 (dT <= 1°C), then the (IR1_A_, IR1_B_) = (0, 0);

if the IR1 scale was 1 (1°C<dT <= 2°C), then the (IR1_A_, IR1_B_) = (1, 0);

if the IR1 scale was 2 (dT>2°C), then the (IR1_A_, IR1_B_) = (0, 1).

In addition, β_i _values (where i = 0, 1_A_, 1_B_, 2~5) indicated the regression coefficients for each IR sign and age factor under this multivariate regression model. IR2 to IR5 in this model indicated the scales for IR2 to IR5 signs of each lesion scored by the interpreting radiologists.

Further, we substituted the age and IR scales read by the radiologists into the age-adjusted multivariate regression model, thus obtaining the ln(OD) of each lesion. The cut-off values were derived and selected from the ln(OD) of this model. Under a given cut-off point or threshold, the ln(OD) of a lesion higher than or equal to the threshold implied a positive IR-test. For each given cut-off point, we also estimated the corresponding sensitivity, specificity, positive predictive value (PPV), negative predictive value (NPV), and Youden's Index (Index = sensitivity+specificity-1) [17,18]. The selection of cut-off points in our study was based on the criteria shown below:

For the cut-off values with sensitivity >90%, we selected one of them with highest corresponding specificity as the lowest cut-off value. Among those cut-off values with specificity>90%, we selected the one with highest corresponding sensitivity as the highest cut-off value. The cut-off value with the highest Youden's Index was also included in the analysis and was regarded as the most optimal cut-off point in our study. We also included other cut-off values between the highest and lowest cut-off values mentioned above, and a total of 5 cut-off values were taken for analysis.

We also evaluated the BI-RADS^® ^(Breast Imaging Reporting and Data System) categories [19] of lesions on mammography and ultrasound, and the corresponding true disease status (the histopathologic results). If the patient received only mammography or ultrasound, the BI-RADS^® ^category of the lesion was determined by one of the imaging modalities; if the patient received both examinations, the BI-RADS^® ^category was given based on higher concern between the two imaging modalities. Based on the most optimal cut-off point and the cut-off value with the highest sensitivity we selected, the corresponding sensitivity, specificity of IR imaging in each BI-RADS^® ^category were also estimated. Since breast cancer may be inherently a life-threatening disease and the false-negativity (1-sensitivity) would cause severe consequences in clinical practice of breast imaging, therefore, we also included the selected cut-off value with highest sensitivity for detailed analysis.

Based on the age-adjusted multivariate model, the ln(OD) values of malignant and benign foci for all lesions were compared using Student's *t*-test. For the lesions with mammographic findings available for correlation, we categorized them into three major types of mammographic findings, that is, microcalcifications, microcalcifications associated with mass (or architectural distortion, focal asymmetry), or noncalcified lesions. We also investigated if there was any difference in ln(OD) values between malignant and benign lesions for different types of mammographic findings using Student's *t*-test or non-parametric Mann-Whitney U test.

We further stratified all lesions into three categories according to the pathologic size, that is, size larger than or equal to 2 cm, size less than 2 cm but not less than 1 cm, and size less than 1 cm across the largest diameter. We compared the ln(OD) values for lesions between benign and malignant lesions for these three categories by Student's *t*-test or Mann-Whitney U test, in order to investigate the validity of the age-adjusted multivariate model in lesions with different size categories.

In our study, a *p *value less than 0.05 was considered to show statistical significance.

## Results

In our study, a total of 298 lesions associated with 276 patients were excised with histopathological correlation. The clinical, conventional imaging, and pathological data are listed in Table 1. Of those, 174 lesions were malignant (DCIS, 22; invasive carcinoma, 152) and 124 lesions were benign (including 7 ADH, 2 ALH and 3 papillary lesions identified as high-risk lesions). The patients with malignant lesions tended to be older than those with benign lesions (*p *< 0.0001). The mean lesion size or extension of malignant lesions on preoperative conventional imaging studies and at surgical pathology was significantly larger than that of the benign lesions (Table 1). There were 215 lesions with available mammographic examinations for correlation. Of the lesions showing mammographic finding of microcalcifications, most of them were benign. Most of the lesions with the finding of microcalcifications with mass or noncalcified finding were malignant (Table 1).

**Table 1:** Clinical and conventional imaging findings.

Basic findings	Malignant	Benign	*p *value
Patient (lesion) number	165 (174)	111 (124)	
Age of patients (years)	54.3 (SD 11.1)	45.4(11.2)	<0.0001^#^
Mammographic findings	122^§^	93	
Microcalcifications	28	77	<0.0001^##^
Calcifications+mass*	40	3	
Noncalcified lesion	51	13	
Available ultrasound findings relevant to the lesion sites	125^§§^	64	
Imaging size**, cm (range)	2.83 (0.3-11)	1.94 (0.24-10)	<0.0001^#^
Pathologic size, cm (range)	2.88 (0.3-12)	2.15 (0.2-7.0)	0.001^#^

*Lesions revealing calcifications associated with mass, focal asymmetry or architectural distortion.

**Lesion size determined by the largest diameter between mammography and breast ultrasound.

^§^: 3 cancerous lesions were not shown on mammograms (false-negative).

^§§^: 2 cancerous lesions were negative on ultrasound.

^# ^Student's *t*-test;^ ##^: Fisher's Exact Test.

Univariate logistic regression analysis of the diagnostic IR signs and the final results are shown in Table 2, and the malignant result was significantly associated with higher IR scores for all IR signs. However, for the age-adjusted multivariate regression analysis, only IR1, IR4 and IR5 remained statistically significant; and IR2 and IR3 turned out to be non-significant (Table 2). The Hosmer and Lemeshow test revealed a non-significant *p *value (p = 0.496), suggesting that the age-adjusted multivariate regression model was an acceptable, fitted model.

**Table 2:** Analysis of IR signs and the final results by univariate and age-adjusted multivariate logistic regression.

IR signs scores		M	B	p*	Univariate ^#^OR(95%CI)(p**)	^##^Multivariate ^#^OR(95%CI)(p**)
IR1 0		68	93	<0.0001	1	1
1		66	27		3.3(1.9-5.8)(<0.0001)	1.5(0.7-2.9) (0.29)
2		40	4		13.7(4.7-40.0)(<0.0001)	6.8(2.0-23.5)(0.003)
IR2 0		70	78	0.0001	1	1
1		104	46		2.5(1.6-4.0)(0.0001)	1.2(0.6-2.3) (0.61)
IR3^&^		174	124	-	1.4(1.2-1.7)(<0.0001)	1.2(0.95-1.4)(0.13)
IR4 0		140	114	<0.01	1	1
1		34	10		2.8(1.3-5.8)(<0.01)	2.8(1.1-6.8)(0.024)
IR5 0		38	77	<0.0001	1	1
1		136	47		5.9(3.5-9.8)(<0.0001)	2.8(1.4-5.8)(0.005)

M: malignant lesions; B: benign lesions; CI: confidence interval.

^#^OR: odds ratio; ^##^multivariate: age-adjusted multivariate regression model.

^&^: continuous variable.

*p*: *p *value estimated by *Chi-square test, **logistic regression analysis.

**Figure 5:**
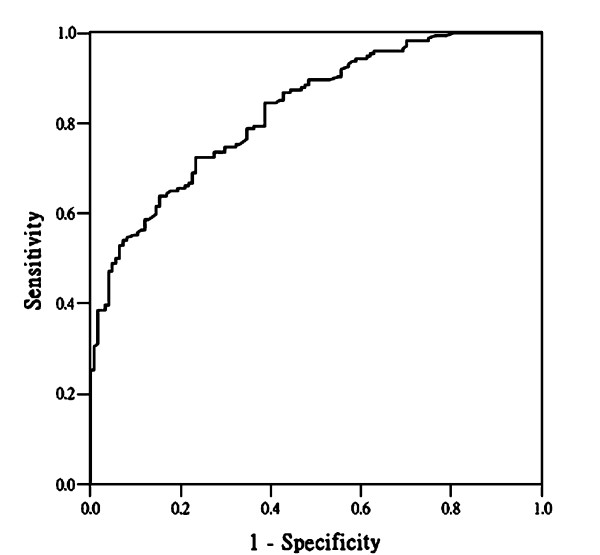
**The ROC of an age-adjusted multivariate regression model**. AUC = 0.828.

When investigating the diagnostic performance of each individual IR sign (Table 3), the IR5 revealed a highest AUC value among the five IR signs. However, the AUC of the age-adjusted multivariate regression model was 0.828 (Figure 5), which was higher than the AUC values of any of the individual IR signs; and its lower bound of 95% CI was higher than the upper bound of the 95% CI of the AUC of all of the individual IR signs (Table 3). The intercept and regression coefficients of the age-adjusted multivariate regression model are shown in Table 3. The selected cut-off values from the age-adjusted multivariate regression model with the derived sensitivities, specificities, PPV, NPV and Youden's Index are presented in Table 4. The highest Youden's Index was 0.49 (most optimal cut-off point), when the cut-off value was 0.3 with a sensitivity of 72.4%, specificity of 76.6%, PPV of 81.3% and NPV of 66.4% for the overall study population. According to the selection criteria we established, the lowest cut-off value of this model was -0.72, with its corresponding sensitivity 92.0%, specificity 44.3%, PPV 69.9% and NPV 79.7%; the highest cut-off value was 1.27, and its corresponding specificity was 94.3% at a sensitivity of 50.0%, PPV 92.6%, NPV 57.4%. The estimation of ln(OD) value for a malignant lesion and the corresponding IR, mammographic images are illustrated in Figures 3, 4.

**Table 3:** AUC values for each IR sign and for an age-adjusted multivariate logistic regression model.

model	AUC	95%CI
Univariate		
IR1	0.699	(0.639, 0.758)
IR2	0.613	(0.549, 0.678)
IR3	0.674	(0.611, 0.736)
IR4	0.557	(0.492, 0.623)
IR5	0.701	(0.640, 0.763)
Multivariate		
(age-adjusted)	0.828	(0.783, 0.873)

Multivariate: age-adjusted multivariate regression model, which is shown as below:

ln(odds) = ln(OD) = α+β_0_(Age)+β_1A_(IR1_A_)+ β_1B_(IR1_B_)+β_2_(IR2)+β_3_(IR3)+β_4_(IR4)+β_5_(IR5)

= -5.463+0.0872(Age)+0.3783(IR1_A_)+1.9157(IR1_B_)+0.1728(IR2)+0.1578(IR3)+1.0278(IR4)+1.0363(IR5).

**Table 4:** The cut-off points, sensitivity, specificity and Youden's Index derived from an age-adjusted multivariate regression model.

Cut-off*	Sen (%)	Spe (%)	Youden	PPV (%)	NPV (%)
1.27	50.0	94.3	0.443	92.6	57.4
0.82	62.6	84.7	0.473	85.2	61.8
0.30^#^	72.4	76.6	**0.490	81.3	66.4
-0.30	85.6	57.3	0.429	73.8	74.0
-0.72	92.0	44.3	0.363	69.9	79.7

*Cut-off: cut-off values, derived from ln(odds), which is the natural logarithm of the odds (OD) from the age-adjusted multivariate regression model. A lesion with a ln(OD) value higher than or equal to a given cut-off point is regarded as IR test-positive.

Sen: sensitivity; Spe: specificity; Youden: Youden's Index = sensitivity+specificity-1.

PPV: positive predictive value; NPV: negative predictive value.

** highest Youden's Index value (^#^the most optimal cut-off point we selected from the model).

The cut-off value was -3.51 when the sensitivity was 100% and the specificity 0%; it was 5.47 when the sensitivity was 0.57% and specificity 100% (not shown in the table because they didn't meet the selection criteria of cut-off points in our study).

When we used the ln(OD) value of 0.3 as the most optimal cut-off point, the corresponding sensitivity, specificity in each BI-RADS^® ^category are shown in Table 5. For the BI-RADS^® ^category 3 lesions, IR imaging correctly identified the only 1 cancerous lesion (sensitivity 100%) with a specificity of 75% (6/8). In BI-RADS^® ^category 4B findings, the IR imaging correctly identified highest proportion of true-negative lesions (specificity 84.6%, 33/39) compared with other categories but the sensitivity (51.5%, 17/33) was somewhat lower than the other categories (Table 5).

**Table 5:** The correlation of BI-RADS^® ^categories on conventional imaging and the corresponding diagnostic performance of IR imaging based on the two cut-off points (0.30 and - 0.72)

^†^BI-RADS^®^ (^‡^n = 281)	True status (n)	Cut-off (1) ln(OD)		Sen(%)	Spe(%)	Cut-off (2) ln(OD)		Sen(%)	Spe(%)
		>=0.30	<0.30			>= -0.72	<-0.72		
3 (n = 9)	B (n = 8)	2	6	100	75.0	4	4	100	50.0
	M(n = 1)	1	0			1	0		
4A (n = 71)	B(n = 60)	15	45	54.5	75.0	38	22	81.8	36.7
	M(n = 11)	6	5			9	2		
4B (n = 72)	B (n = 39)	6	33	51.5	84.6	17	22	84.8	56.4
	M(n = 33)	17	16			28	5		
4C (n = 95)	B (n = 10)	4	6	74.1	60.0	6	4	92.9	40.0
	M (n = 85)	63	22			79	6		
5 (n = 34)	B (n = 2)	2	0	81.3	0	2	0	100	0
	M (n = 32)	26	6			32	0		

^†^BI-RADS^®^: BI-RADS^® ^category, an integrated BI-RADS category assessment based on conventional imaging (mammography and/or ultrasound), according to higher concern in any of or the two combined imaging modalities.

^‡^There were 17 lesions from 17 patients having outside mammography and/or ultrasound and the detailed statements of BI-RADS^® ^categories were not obtained, and thus leaving 281 lesions for evaluation.

BI-RADS^® ^Category 3: probably benign finding.

BI-RADS^® ^Category 4A: a lesion with low suspicion level for malignancy.

BI-RADS^® ^Category 4B: a lesion with intermediate concern for malignancy.

BI-RADS^® ^Category 4C: a lesion with moderate concern for malignancy.

BI-RADS^® ^Category 5: a lesion which is highly suggestive of malignancy.

B: benign (including high-risk lesions); M: malignant.

Cut-off (1): the cut-off value of 0.30; cut-off (2): the cut-off value of -0.72.

ln(OD)>= the selected cut-off value indicated IR-test positive, and < the cut-off value indicated IR-test negative; ln(OD): ln(odds).

Sen: sensitivity = (true-positive number)/(the number of malignant disease status); while true- positive indicated IR-test positive as well as disease positive (malignant).

Spe: specificity = (true-negative number)/(the number of benign disease status); while true-negative indicated IR-test negative as well as disease negative (benign or high risk lesions).

On the other hand, when we used the -0.72 as the cut-off point, the sensitivity in each BI-RADS^® ^category increased while the specificity decreased when compared with the counterparts using 0.3 as the cut-off point (Table 5). Of them, in BI-RADS^® ^category 3 findings, the IR imaging again can identify the only one cancerous lesion but the specificity dropped to 50% (4/8); the corresponding sensitivity of the BI-RADS^® ^category 4A findings was lowest (81.8%, 9/11) among all categories with a specificity of 36.7% (22/60). The specificity in BI-RADS^® ^category 4B (56.4%, 22/39) was higher than other categories with a sensitivity of 84.8% (28/33), and the sensitivities in BI-RADS^® ^categories 4C and 5 were higher than 90%.

For all of the 298 lesions, the mean ln(OD) value of the malignant lesions (1.40) based on the age-adjusted multivariate model was higher than that of benign lesions (mean ln(OD) = -0.58) with statistically significant difference (*p *< 0.0001, Student's *t*-test). We further estimated the ln(OD) values for lesions with mammographic findings available for correlation. For the lesions showing microcalcifications on mammograms, the mean ln(OD) value of malignant lesions (mean = 0.73) was significantly higher than that of benign lesions (mean = -0.4; *p *= 0.001, Student's *t*-test). For the lesions revealing calcifications with mass, the malignant lesions tended to have higher ln(OD) values (median = 1.01) than benign lesions (median = -0.63) with statistically significant difference (*p *< 0.01, Mann-Whitney U test). For lesions with noncalcified findings, the malignant lesions again tended to have higher ln(OD) values (median = 1.35) than benign lesions (median = 0.23; *p *= 0.002, Mann-Whitney U test).

For the lesions stratified by different pathologic size categories, the ln(OD) values of the malignant lesions tended to be higher than those of benign lesions with statistically significant difference in all three categories (Table 6).

**Table 6:** The ln(odds) values (ln(OD)) between benign and malignant lesions stratified by size

	M^†^	B^‡^	*p*
size*(cm)>= 2			
n	111	51	
ln(OD)	1.65^a^	-0.61^a^	< 0.001^#^
1 <= size*(cm)<2			
n	46	27	
ln(OD)	1.20^a^	-0.74^a^	< 0.001^#^
size*(cm)<1			
n	17	46	
ln(OD)	0.36^a^	-0.62^m^	< 0.028^§^

^†^M: malignant;^ ‡^B: benign (including high-risk lesions); ln(OD): ln(odds).

*size: determined by the pathologic size (across the largest diameter).

^a^: Average, mean ln(OD).

^m^: Median ln(OD).

^#^: Student's *t*-test; ^§^: Mann-Whitney U test.

## Discussion

Though infrared imaging of the breast is not widely used for various reasons, it has been reported to show promising results in some series [6,7,9-12,15,20]. Parisky, et al [6] used computerized IR imaging for suspicious findings on mammograms, and reported that IR imaging featured a relatively high sensitivity (97-99%) and negative predictive value (95-99%) and thus can differentiate benign from malignant lesions reliably. However, the reported specificity in this series ranged from 14 to 18%, which was relatively low. Keyserlingk, et al [7] reported that the sensitivity of mammography alone was 85% and that of combined modalities of digital IR, mammography was 95%. Thus, digital IR imaging can provide additional information for breast lesion diagnosis. There were some authors from different series who used an artificial neural network, computer software, or segmentation technique for breast cancer detection, monitoring of treatment response, and for establishing an interpretive model [3,5,11,12,15,20]. However, though digital IR imaging uses the combined criteria of temperature and vascular pattern for diagnosis, the diagnostic criteria are still somewhat variable according to the aforementioned series [3,5,7,11,12,15,20]. In our study, for a subpopulation with diagnostic purpose, we tried to establish an age-adjusted multivariate regression model to predict breast cancer disease status based on IR findings, and to investigate the diagnostic performance of IR imaging based on this model.

For univariate analysis of the different diagnostic IR signs in our study, we found that there was a significant association between malignancy and higher IR scores for each IR sign, and the IR5 (asymmetric vascular pattern at lesion site compared to the contralateral side) showed a higher AUC value than other signs. This was not surprising, since IR imaging of the breast was designed to detect temperature elevations of the tumor, and the site with elevated surface temperature will indeed cause an asymmetric thermographic pattern. Therefore, though the IR1 sign has a somewhat similar implication as IR5, it is a quantitative measure, while IR5 is a morphologically descriptive sign. In addition, for a better comparison of the lesion site with the contralateral breast, we excluded women who had breast surgery previously, to eliminate the inherent asymmetric thermographic pattern due to parenchymal loss after partial or total mastectomy.

In age-adjusted multivariate regression analysis, the malignant result was associated with higher IR scores, especially for IR1, IR4 and IR5 when adjusted for other factors. Indeed, this model revealed a better diagnostic performance when compared with each IR sign from univariate analysis, based on a diagnostic population in our study. Though there were some series reporting the diagnostic value of IR imaging for breast cancer screening [1,4,9,21,22], and abnormal IR imaging of the breast was reported to be a cancer risk predictor [9], IR imaging has not been routinely used for screening purpose. Our study, like some of other series [6,7,11], investigated the diagnostic efficacy of IR imaging for specific groups with inconclusive or suspicious findings on conventional imaging modalities (mammography or ultrasound). In addition, most of these reported series combined the mammographic, clinical breast examination and IR together to discuss the diagnostic values of IR as an adjunct tool. But these series seldom included ultrasonographic correlation [6-8,11]. However, in our study, there was a considerable proportion of participants that had mammographic and ultrasonographic correlations, and a combined analysis of both imaging modalities would provide a more convincing diagnostic result than if only one of the modalities was available. Therefore, we designed our study so that the interpreting radiologists were informed of only the lesion site and size on conventional imaging modalities when reading the IR images, and were blinded to the clinical history and detailed mammographic and ultrasonographic findings, and the pathological results. We established this protocol to ensure a more objective reading of IR images. This allowed us to establish an interpretive model for IR imaging of the breast with less bias. After establishing an appropriate interpretive model, it would be possible to apply this model or similar methodology to other groups, such as a screening population.

In our study, the cut-off values of the IR imaging were estimated from the age-adjusted multivariate regression model, which also derived the AUC of the ROC curve for diagnostic performance evaluation, and the various sets of sensitivity and specificity values can be inferred from different cut-off values. There is always a trade-off between sensitivity and specificity, since a higher sensitivity will be accompanied with a lower specificity and vice versa. The above observations also apply to the analysis of PPV and NPV. As can be seen in Table 4, cut-off values with higher PPV and specificity correspond to a lower NPV and sensitivity, and vice versa. The strategy of which arm should be stressed more depends on the purpose of the test and the target population enrolled [17,18,23]. Under this consideration, the cut-off value with the highest Youden's Index might not be the most optimal threshold value [17,18,23]. For the lowest selected cut-off point (sensitivity 92.0% and specificity 44.3%) in our study, it showed a relatively high sensitivity and an acceptable specificity when compared with other reported series [6,11]. However, in BI-RADS^® ^category 3 findings, the cut-off point with highest Youden's Index (cut-off value 0.3) yielded a high sensitivity and moderate specificity (75%), but the specificity dropped to 50% when using the -0.72 as the cut-off point. Therefore, for the IR imaging, the cut-off value of 0.3 was a more optimal cut-off point for probably benign findings as an adjunct role in diagnostic breast imaging. And for the category 4A and 4B findings, though the sensitivities using the -0.72 as the cut-off point increased as compared with the cut-off point of 0.3, the values (81.8% in 4A and 84.8% in 4B; Table 5) were still not satisfactory in a diagnostic population. However, if we read the IR imaging after viewing the detailed mammographic and ultrasound images, the diagnostic performance may be different and should be likely elevated somewhat compared with our current study design. In that situation, the clinical role of IR imaging may be more enhanced at the help with decision making when the mammography or ultrasound shows ambiguous findings.

In our study, for the lesions with mammographic findings available for correlation, we found that the malignant lesions tended to have higher ln(OD) values (ln(odds) values) than benign lesions in all three types of mammographic findings.

When we compared the ln(OD) values between the benign and malignant lesions stratified by different pathologic size categories, we found that the malignant lesions tended to have higher ln(OD) values than benign lesions in all three categories, even for lesions less than 1 cm and 2 cm in size. The findings further ensure the validity of application of the age-adjusted multivariate model to lesions with smaller tumor size.

There are some limitations to our study. First, as we stated previously, our study participants were for diagnostic purpose, and we interpreted the IR images referring to the lesion site and size, that is, a targeted interpretation. Therefore, currently we have not yet documented the diagnostic value of our model for screening purposes. Second, for the purpose of keeping the statistical efficiency of the model analysis, we categorized 12 high-risk lesions into the benign group due to the limited sampling size, and we didn't further categorize the malignant lesions into non-invasive and invasive, or different grades of carcinomas. Therefore, the diagnostic performance of IR imaging in high-risk lesions or different pathologic grades of carcinomas may not be known. Finally, we excluded patients having a past history of breast surgery to reduce the interpretation bias caused by asymmetric thermographic pattern due to parenchymal loss, as we mentioned above. Thus, we haven't applied our interpretative model to post-operative breasts.

## Conclusions

In conclusion, IR imaging of the breast is a noninvasive diagnostic examination. We established an age-adjusted multivariate logistic regression model under a specific clinical setting for diagnostic purpose. However, its values for post-operative breasts, for screening populations, and for high-risk lesions or different grades of breast carcinomas have not been verified in our study and should be further investigated in the future. Further, from our study, it has not yet been proven whether the IR imaging would reliably avoid unnecessary biopsy for suspicious findings on mammograms and breast ultrasound.

## Competing interests

The study was supported by AG Digital Technology Corporation, Taipei, Taiwan.

The organization supplied the thermographic machine and funding of the study. However, the article-processing charge is not supported by the organization.

## Authors' contributions

JW participated in the design of the study, imaging interpretation and carried out the statistical analysis, drafted and revised the manuscript. KJC participated in the design of the study, the acquisition of the data, and helped to revise the manuscript. CYC participated in the design of the study, imaging interpretation and carried out the statistical analysis, and revised the manuscript. KLC participated in the statistical analysis and helped to revise the manuscript. YST participated in the design of the study, the acquisition of the data, and helped to revise the manuscript. YMW participated in the design of the study, the acquisition of the data, imaging interpretation, and helped to revise the manuscript. YCT participated in the acquisition of the data and helped to revise the manuscript. TTFS participated in the design of the study, carried out the statistical analysis, drafted and revised the manuscript. All authors read and approved the final manuscript.
